# Misdiagnosis of type 1 diabetes mellitus

**DOI:** 10.1186/1687-9856-2013-S1-P23

**Published:** 2013-10-03

**Authors:** Noor Shafina Mohd Nor, Muhamad Yazid Jalaludin, Fatimah Harun

**Affiliations:** 1 Faculty of Medicine, University Teknologi MARA, Selangor, Malaysia; 2 Faculty of Medicine, University Malaya, Kuala Lumpur, Malaysia

## Introduction

In the past type 1 Diabetes Mellitus is the commonest type of diabetes affecting children in Malaysia. Nevertheless, diagnosis of type 2 Diabetes Mellitus is increasingly being made due to the rising prevalence rate of obesity amongst children. This contributes to the difficulty in making the right diagnosis at initial presentation if the clinical features that distinguish the two overlap.

## Case

An 8 ½-year-old Indian boy is reported here to have been mistakenly diagnosed as type 2 Diabetes Mellitus. He initially presented with a 2-day history of polyuria, polydipsia and nocturia. Initial screening of random blood glucose (RBG) at a local pharmacy reported the blood glucose (BG) as high (27mmol/L). He then presented to the emergency department where the BG was 25.1mmol/L without metabolic acidosis (pH 7.404, bicarbonate 23.0mmol/L). Serum ketones was 0.8 mmol/L. Paternal and maternal grandparents have type 2 Diabetes Mellitus diagnosed in adulthood. He was overweight with BMI of 19.5kg/m^2^ (90^th^ perdentile). Examination revealed acanthosis nigricans on the neck and the axillae. An initial diagnosis of type 2 Diabetes Mellitus was made. The HbA1c was 11.2% and C-peptide 0.8 ng/mL (Normal 0.9-4.0 ng/mL). He was started on Metformin 250 mg daily with the aim of slowly increasing the dose. His BG continued to be between 18.8mmol/L and 27.3mmol/L pre-meals with pre-breakfast BG around 13 to 14mmol/L. He continued to have ketonemia (serum ketones between 0.5 and 1.3 mmol/L) without metabolic acidosis. Hence, basal subcutaneous insulin was added (0.4 unit/kg/day) 3 days after starting Metformin. His BG improved and he was discharged home. On review four weeks later, he had few episodes of mild hypoglycaemia with Metformin 500 mg BD and Glargine 8 units (0.25 units/kg/day) at bedtime. His diagnosis was revised to type 1 diabetes mellitus as his auto-antibodies were markedly elevated i.e. anti-Glutamic Acid Decarboxylase (GAD) >2099.75U/ml (<10 IU/ml), anti-Islet Cell (ICA) 22.06 (<0.7) and anti-IA2 153.91U/ml (<10 IU/ml).

## Conclusion

Making the correct diagnosis of diabetes mellitus in children based solely on clinical phenotype i.e BMI and presence of acanthosis nigricans is no longer reliable. Additional laboratory tests namely C-peptide and elevated auto-antibodies and response to initial treatment are essential to aid in our clinical judgement and correct treatment.

